# Impact of 3D Printing on the Overall Project Success of Residential Construction Projects Using Structural Equation Modelling

**DOI:** 10.3390/ijerph20053800

**Published:** 2023-02-21

**Authors:** Ahsan Waqar, Idris Othman, Juan Carlos Pomares

**Affiliations:** 1 Department of Civil & Environmental Engineering, University Technology PETRONAS, Seri Iskandar 32610, Malaysia; 2 Civil Engineering Department, University of Alicante, 03690 Alicante, Spain

**Keywords:** 3D printing, overall project success (ops), public health, environmental protection, residential construction in Malaysia

## Abstract

After a decade of research and development, 3D printing is now an established technique in the construction sector, complete with its own set of accepted standards. The use of 3D printing in construction might potentially improve the outcome of the project as a whole. However, traditional strategies are often used in the residential construction industry in Malaysia, which causes serious public safety and health issues along with a negative impact on the environment. In the context of project management, overall project success (OPS) has five dimensions, such as cost, time, quality, safety, and environment. Understanding the role of 3D printing in relation to OPS dimensions in Malaysian residential construction projects would allow construction professionals to adopt 3D printing more easily. The aim of the study was to find the impact of 3D construction printing on OPS while considering the implications for all five dimensions. Fifteen professionals were interviewed to first evaluate and summarise the impact factors of 3D printing using the current literature. Then, a pilot survey was conducted, and the results were checked using exploratory factor analysis (EFA). The feasibility of 3D printing in the building sector was investigated by surveying industry experts. Partial least squares structural equation modelling was used to investigate and validate the fundamental structure and linkages between 3D printing and OPS (PLS-SEM). A strong correlation was found between 3D printing in residential projects and OPS. Highly positive implications are indicated by the environmental and safety dimensions of OPS. Malaysian decision-makers may look to the outcomes of introducing 3D printing into the residential construction industry as a modern method for increasing environmental sustainability, public health and safety, reducing cost and time, and increasing the quality of construction work. With this study’s findings in hand, construction engineering management in Malaysia’s residential building sector might benefit from a deeper understanding of how 3D printing is used for improving environmental compliance, public health and safety, and project scope.

## 1. Introduction

A construction project’s success is determined by five essential elements that must be present in the finished result for it to be considered a success. These essential elements are cost reduction, time management, improvement in public health and safety, and environmental sustainability [1,2]. Cost, time and quality (CTQ) are commonly indicated as scope dimensions. Construction project success is critically dependent on improving economic, environmental, and social sustainability. It is the reason that existing studies advocated focusing on cost, environmental compliance and improvement in public well-being [3,4]. It is also one of the major reasons why the construction industry is always improving and new technologies are being adopted. The construction sector has been driven to embrace modern procedures and tools such as digital fabrication to keep up with the demands of current architectural design for flexibility, complexity, high performance, intricacy, customisation of material, and technology [5]. Tay et al. and Yang et al. [6,7] argued that inefficient production methods in the residential construction sector have been advocated for replacement by the use of automation in the construction industry. A shift to this digital architecture paradigm is expected to have far-reaching positive effects on the quality of human-made structures. Consequently, it is incumbent upon architects to design fully automated production methods that promote ideals such as equity, sustainability, democracy, diversity, and inclusion [8,9]. Subrin et al. and Tobi et al. [10,11] stated that understanding the shifts brought about by technological developments in the architectural sector may increase the efficacy of architectural education, stimulate novel strategies for design and construction, and influence the course of future research. Safety has always remained a significant challenge for the construction industry, as it can be compromised if a sustainable contribution is not made through modern technologies [12,13]. Hundreds of workers die worldwide, even on a residential scale, because of ignorance of safety standards and certain limitations of traditional construction methods adopted [14,15,16]. In accordance with Antreas and Piromalis [17], this is the reason that makes the residential construction sector significant enough to be improved with advanced technologies. In addition to manufacturing, 3D printing has several important applications in various fields, as stated by Kantaros and Kondiah et al. [18,19]. In regenerative medicine, 3D printing is used to create biocompatible scaffolds that can support the growth of new tissues and organs, offering new treatment options for patients with injuries or diseases [20,21]. In the aerospace industry, 3D printing is used to manufacture lightweight parts with complex geometries, improving fuel efficiency and reducing emissions. Other applications of 3D printing include creating prosthetic limbs, architectural models, and customised dental implants [22,23].

In three-dimensional printing, the material is deposited in layers using a computer model as a blueprint. Software, hardware, and raw materials are the foundations of 3D printing. The first 3D printer was invented in 1983 by Charles W., and it has since become one of the fastest-growing industries [14,15,16]. A century ago, this technology was exceedingly difficult to operate and pricey. Three-dimensional printers have grown increasingly ubiquitous in industrial settings and have progressively found their way into daily life [24]. According to E. Ali and Korolev et al. [25,26], when talking about 3D printing’s applications in manufacturing, the phrases “3D printing” and “additive manufacturing” are sometimes used interchangeably. When 3D printing technology advances, we will be able to use it to fabricate dwellings. Complex structures may be constructed more sustainably and with less material waste if this method is used. In addition to its various benefits, 3D printing may be included at any stage of the design process, from the creation of preliminary idea drawings to the construction of fully realised houses.

Yang et al. stated that building using a 3D printer eliminates the need for mould support and removal, increasing material efficiency and reducing concrete resource waste [27]. Time frame for building: Due to the enhanced efficiency of 3D printing technology compared to traditional techniques, the construction time may be cut in half, and the payback period may be reduced. Three-dimensional construction printing technology has also shown positive implications regarding maintaining safety on residential construction sites. During the printing process, little noise, dust, and harsh vibrations are produced. Buchanan and Gardner and Elfatah [13,28] argued that the computer-controlled, layer-by-layer construction method helps minimise room for human error and ensures a superior final product [13,28].

As 3D printing can be tailored to specific requirements, stockpiles can be minimised; single-piece production eliminates the need for assembly, and the time between printing and delivery is drastically cut down [29,30,31]. Beneficial in extreme crises Markin et al., Ji et al. and Holt et al. [32,33,34] found that the phrase “extreme environment” is sometimes used to denote that traditional manufacturing processes are no longer applicable and that human activity is impeded when addressing an environment defined by harsh conditions, such as weightlessness or sub-zero temperatures. Three-dimensional printing has the potential to completely alter the construction industry. It is true that 3D printing might significantly cut down on construction expenses, but the expensive cost of printers is a major downside. Hossain et al. and Jawad et al. [35,36] argued that the intricacy of 3D printing software necessitates the use of trained professionals. According to the available data, the electricity consumption of 3D printers is much higher than that of conventional methods, by a factor of 100 [37,38].

The high price of the technology is one of the challenges that 3D printing offers to the construction sector. It is unclear if 3D printing will lead to price increases or decreases. El-Sayegh et al., Koc et al. and Shahrubudin et al. [39,40,41] stated that the costs associated with switching to 3D printing from more traditional methods of production fall into two main categories. Time is the first factor to consider. A better-quality surface finish requires more time and effort to produce, increasing the related labour cost [42]. The optimization cost is a secondary criterion. Costs will increase due to the additional time and effort required for optimization, which might lead to a more complex construction than is required [43,44]. However, materials, labour, and infrastructure are the three most obvious expenditures from the perspective of the entire cost structure of a building. With its automated process, 3D printing will reduce construction costs by decreasing the necessity for, and price of, human labour [37,45], as the 3D printing system generates an easier and simpler construction with limited human labour. The construction safety risk reduces, so it is no longer necessary to implement collective or individual-level safety measures. Serious accidents involving major injuries, such as falling from heights, can also be avoided with the use of 3D construction printing [1,2]. Ultimately, it shows positive implications for public health and safety.

The use of 3D printers in the building industry is not new, and it is only expected to increase in popularity. Iuliano et al. and Tahmasebinia et al. stated that, due to the rapidity of the projects and the lower cost, an additive building has the potential to disrupt the traditional construction business [46,47]. Corporate activities that have negative effects on the environment and society are coming under closer scrutiny, so technology improvements such as 3D printing are quite welcome. Business enterprises have an essential role in modern society, and one aspect of this role is to conduct themselves ethically. Cascone et al. and García-Alvarado et al. [48,49] argued that with help from the government and numerous partners, 3D printing is likely to keep improving, allowing for faster and cheaper house building in Malaysia. There is a chance that in the near future, the residential sector of Malaysia may adopt a prefabrication or on-site 3D printing method for buildings that would dramatically cut carbon emissions [50,51]. Historical data reveal that architects and the construction industry are willing to adopt and improve upon this innovation [52,53,54]. Many authors have produced overviews of this technology and its consequences and uses in construction, and the number of these publications is growing. Shahzad et al., Mascarenhas and Makhoul, and Kazemian and Khoshnevis [55,56,57] stated that, while these analyses cover a wide range of topics, they often zero in on one facet of technology and how it is used. However, the present level of study lacks the systematisation necessary to offer a full overview of the technology’s uses and influence in the residential building sector of developing nations such as Malaysia. 

Despite the lack of information, further study is needed to determine how 3D printing affects new home construction in Malaysia. It has been observed that the lack of understanding of 3D printing among Malaysian residential building stakeholders prevents its use in ensuring overall project success (OPS) as measured by cost, time, quality, public health safety and environment. Despite this basic familiarity, research shows that practitioners require a deeper understanding of the implementation notion at hand. According to Besklubova et al., Elias Ali and Pan et al. [58,59,60], while this is an obvious need for the residential construction industry, earlier research on residential 3D building in Malaysia has not made the connection to OPS. A project is regarded as successful if it is finished on schedule, under budget, and to a high enough quality standard as determined by OPS. The risk of 3D construction printing as a new construction technology has been accompanied by a lack of awareness of the more real impact factors that significantly influence the OPS of residential building projects in Malaysia. In Malaysia, where efforts are being made to secure the long-term profitability of smaller-scale building projects, the results of this research are crucial to the growth of the residential construction sector. Since 3D printing has already been shown to be a technology that brings high sustainability to the construction industry, businesses and people in the residential building projects in Malaysia would do well to educate themselves on the current impact elements of adopting the technology. With that in mind, the goal of this research is to examine how 3D printing can influence the OPS of Malaysia’s residential construction sector. The purpose of this research was to examine the link between 3D printing and operating profit margins in Malaysian residential construction projects using partial least square (PLS) modelling.

## 2. Research Model Development

### 2.1. 3D Printing Measurements

Concrete 3D printers are widely used in the modern construction technique known as 3D printing. Multiple academic articles are required on the method to evaluate its structure and methodology [38,61,62]. The use of 3D printing in the building industry has the potential to increase sustainability in various ways. There are five main advantages of using 3D printing in construction, such as, “Function”, “Sustainability”, “Standardization”, “Creativity” and “Credibility”. These are, perhaps, the most far-reaching implications of 3D printing for the construction sector. Table 1 shows the 3D printing impact factors that are identified as being related to project success.

### 2.2. Cost, Time, Quality, Safety, Environment Measurements

A completed building project is now seen as par for the course. This highlights the need for establishing common ground amongst the project’s customers, designers, and consultants over the definition of success. Principles of project management state that timely, cost-effective, and high-quality project completion, along with public health and environmental protection, are the five most important reflective indicators of a construction project’s success. The variables that affect a construction project’s safety and completion on schedule are listed in Table 2.

### 2.3. Connection between 3D Printing and Residential OPS

**Figure 1:**
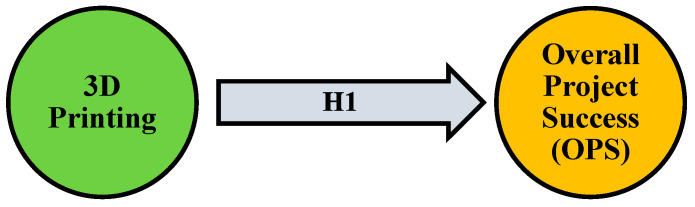
Hypothesised relation between 3D printing and OPS (time, cost, quality, public health and safety and environment).

The overall success of a project is dependent on five important aspects, including cost, time, quality, environment, and safety precautions. If these five conditions are met, every construction project may be deemed a success; hence, the OPS is based on effective time management, regulated cash flow, quality assurance, environmental preservation, and public safety [37,87,91]. It is because of the fact that the construction project’s success should provide benefit to society, the environment, and the economy [44,90,91]. It has been investigated whether or not 3D printing may be used to expedite mundane but necessary building operations. Before declaring the endeavour a success, however, it was necessary to weigh the numerous implications of 3D printing. Three-dimensional printing’s application to the building trades will determine the project’s ultimate success. E. Ali, Korolev et al. and Tan [16,25,26] conclude that advancements in 3D printing of buildings will allow for a higher probability of success in the long run. These days, projects are more complex and need novel approaches that use the synergy of those involved to ensure the project’s success. Elfatah, Lee et al. and Marchment et al. [28,29,31] stated that conflicts may be avoided and participants’ expectations for the project’s success can be bolstered by using 3D printing in construction to resolve and manage their future concerns. According to El-Sayegh et al., Florea et al. and Hossain et al. [36,41,77], in terms of overall project success and project completion, the impact of 3D construction printing on employees and businesses is different from what has been witnessed in the literature thus far. OPS becomes therefore certain when any construction project does not result in any causing injury to workers, increased cost than planned budget, impacted the timeline, reduced quality, and affected the environment. This research is significant because it will contribute to our understanding of 3D building printing and its impact on OPS by shedding light on how these technologies are really put to use. To the best of our knowledge, this is also one of the first empirical studies performed in Malaysia. In this study, 3D printing is an independent variable and OPS is an independent variable. The main hypothesis is, H1: There is a significant relationship between 3D printing and OPS. The paper goes into depth on the impact that different 3D printing methods had on OPS in the Malaysian manufacturing industry, including the planning and implementation stages. The value of a study is shown when it provides theoretical insight into the phenomena being examined, in this case, 3D building printing in a new nationwide environment. The hypothesis of this study relative to the connection between 3D construction printing and residential OPS is given in Figure 1.

## 3. Research Approach

The first stage in developing a theory is to devise a research approach. Ringle et al., The first stage in developing a theory is to devise a research approach. Ringle et al., Savalei and Tarka [92,93,94] stated that, in reality, the theoretical model is only a synopsis of a literature review tailored to the issue at hand, which is then utilised to generate preliminary hypotheses. The three steps of the conceptual modelling method are (1) establishing the model’s components, (2) categorising the constructs, and (3) documenting their relationships. 

Structural equation modelling (SEM) is a statistical technique that allows researchers to test complex theoretical models that involve multiple interrelated variables. SEM is a form of regression analysis that includes both observed and latent (unobserved) variables, and it can be used to analyse both cross-sectional and longitudinal data [95]. The technique allows researchers to test and refine theoretical models, estimate and test direct and indirect effects between variables, and assess the fit of the model to the data.

**Figure 2:**
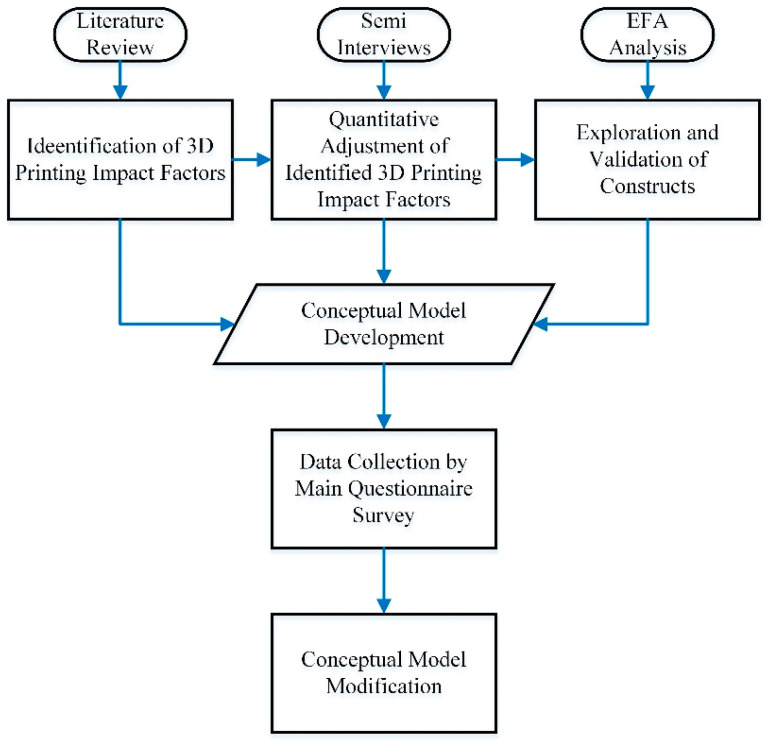
Research methodology flowchart.

The methodology flowchart is presented in Figure 2. Fifteen specialists in the Malaysian residential building industry were contacted to validate and classify the 3D construction impact factors according to Table 1. Zyphur and Pierides [96] stated that it has been shown from prior research that there are just a handful of worldwide drivers impacting 3D printing acceptance and regulatory focus. Therefore, this study included a pair of questionnaires. Before using measuring equipment in the main research, it is standard practice to perform a pilot study to ensure its accuracy. Primary research is the next stage to test this notion.

### 3.1. Pilot Study

The pilot study employed exploratory factor analysis (EFA) to analyse the results of a single survey given to a random selection of Malaysian residential construction firms. Between 150 and 300 samples are required for EFA analysis [97]. According to Igolkina and Meshcheryakov, Shirgaokar and Larsson et al. [98,99,100], it is advisable to limit the number of variables in a factor evaluation to between 20 and 50, since any more than that makes it hard to offer a granular breakdown of the factors. This is because a larger sample size enabled the researchers to use fewer variables. The population size was determined according to the most advanced and locally active residential construction companies (40) and the number of employees in each company (10). This gave a population size of 400, from which the sample size of 200 was determined in accordance with randomised sampling and a 5% margin of error.

### 3.2. Main Questionnaire

**Figure 3:**
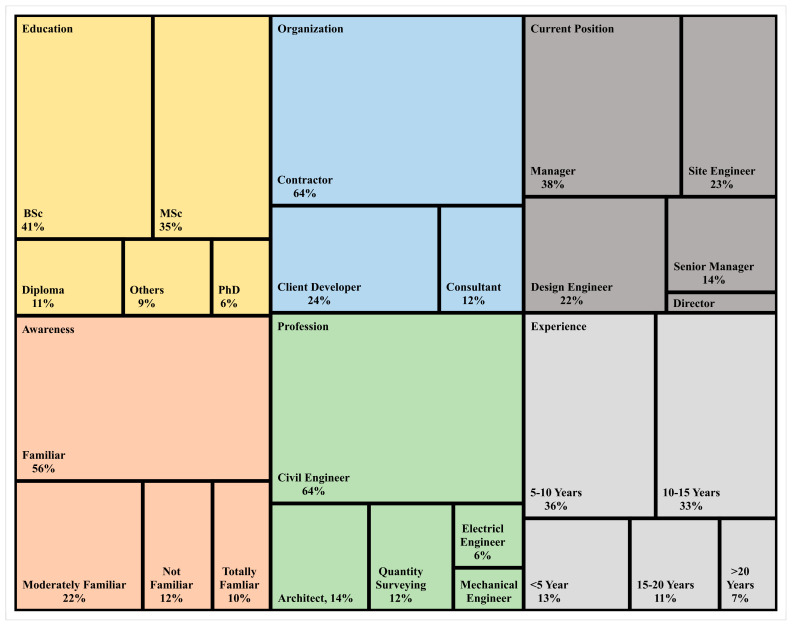
Demographic profile.

The results of the research led to the creation of a standardised cross-sectional survey. The activity categories were revised once the first interviews and EFA evaluation were completed. The Malaysian state of Perak served as the study’s location. In order to assess the impact of 3D printing on the construction industry and residential OPS issues, we solicited responses from a large pool of industry professionals via a primary survey. The four main sections of this questionnaire were: (1) the respondent’s demographic profile; (2) the impact factors of 3D construction printing (Table 1); (3) the overall project characteristics (time, cost, quality, safety, and environment); and (4) open-ended questions to solicit suggestions for additional activities deemed essential by respondents. Figure 3 shows the distribution of the survey to the three key stakeholders: contractors, consultants, and clients. Subspecialties of these careers include those of electrical engineers, architects, quantity surveyor specialists, civil engineers, and mechanical engineers. 

The demographic profile of the participants involved in the study is shown in Figure 3. From an education perspective, 11% had a diploma, 41% had a bachelor’s degree, 35% had a master’s degree, 6% had a PhD, and the remaining 9% had other types of education. From an organisation perspective, there were 64% contractors, 12% consultants, and 24% client developers. From a current working position perspective, 35% were managers, 22% were design engineers, 23% were site engineers, 14% were senior managers, and only 3% were directors. It is observed that 12% of respondents were not familiar, 56% were familiar, 22% were moderately familiar, and only 10% were totally familiar with 3D printing technology in the construction industry. From a profession perspective, 64% were civil engineers, 14% were architects, 12 were quantity surveyors, 6% were electrical engineers, and only 4% were mechanical engineers. From an experience perspective, 13% of participants had less than 5 years, 36% had 5 to 10 years, 33% had 10 to 15 years, 11% had 15 to 20 years, and 7% had greater than 20 years of experience. 

### 3.3. Analytical Approach

To explore how 3D printing in construction affects the success of building projects, four models from the literature were analysed and compared to the best alternative generated by utilising 3D printing to establish a simulation model for successful construction development. Multiple linear regression, system dynamics, structural equation modelling, and artificial neural networks. The link between non-observed variables has barred the regression equation from being applied. This substantially limits the use of the regression equation.

The system’s dynamics could not be exploited as there was no temporal link in the data (i.e., the information is not time-related) (i.e., the information is not time-related). According to Savalei and Tarka [92,93], the fundamental purpose of the research is to analyse how 3D printing of a home building may be utilised to accomplish OPS, and the ANN is undoubtedly a predictive advanced model that does not completely fit the sort of data included in the study. The SEM technique may be used to describe the connection between as many unobservable and observable components as the study’s scope permits. SEM is an efficient approach for controlling typographical mistakes [96,100]. To establish the association between 3D printing in home building and OPS, a model was constructed using SEM in this study. Despite the broad use of hypothesis testing methodologies, SEM has become a well-established non-experimental scientific methodology. Ringle et al. Savalei and Wang and Rhemtulla [93,94,101] have shown this tactic’s efficacy over time. According to Larsson et al., Igolkina and Meshcheryakov and Shirgaokar [98,99,100], as an added bonus, SEM is widely recognised and used as a credible resource for data collection and analysis in the social sciences because of its apparent usefulness in the building industry. Activities within the 3D printing impact factors and OPS were studied using the PLS model, which incorporates both formative and reflecting components, to determine the nature of the interaction between them. The measurement model applying PLS explains the relationships between the idea of 3D construction printing and the observed indicators.

## 4. Results

An EFA was performed to explore the factor structure of 23 important 3D printing influence components. The link was constructed by combining previously established factors of factorability. The Kaiser–Meyer–Olkin (KMO) test is often used to determine whether or not missing linkages between variables are negligible. Ringle et al., Wang and Rhemtulla and Zyphur and Pierides [94,96,101] stated that the lowest possible value of the KMO index for effective factor analysis is 0.6. In addition to the non-identity matrix construction, the sphericity test of the correlation matrix proposed by Bartlett allows for its construction in the identity matrix form. Assuming Bartlett’s sphericity test succeeds, only then can we have faith in the factor analysis (*p* < 0.05). Both the KMO sample sufficiency measure (0.544) and Bartlett’s test seem to be statistically significant. Although variables with fair values (0.3) do not precisely match the factor solution, the initial communalities (estimates of variance when all contributing factors are included) are consistent with the null hypothesis. Based on what we know now, it seems that prehistoric societies had great prosperity. There was no negative loading factor. In the EFA, five of the 20 variables had eigenvalues greater than 1. The five components and their eigenvalues explained 59.418 percent of the total variance.

Four critical components, 3DP.PR1, 3DP.PR2, 3DP.PR3, and 3DP.PR4, made up the last component of inventiveness. Due to their large cross-loadings and loadings below 0.6, the variables 3DP.PR2 and 3DP.PR4 were removed from the analysis; similarly, the variable 3DP.PR2 was not permitted to go to the standardisation phase. Thus, Table 3 provides a rundown of the top five candidates for extraction components in light of 3D printing theory. The statistical validity of the EFA-retrieved variables was assessed. According to Hayward et al. and Savalei [93,102], Cronbach’s alpha values over 0.6 are considered sufficient for newly created measures, with 0.7 being the norm and 0.8 reflecting exceptional reliability. High reliability is statistically indicated by a Cronbach’s alpha score over 0.7. All average set correlations were greater than 0.3, indicating statistical stability among the objects’ internal variables.

### 4.1. Measurement Model (First Order Construct)

**Figure 4:**
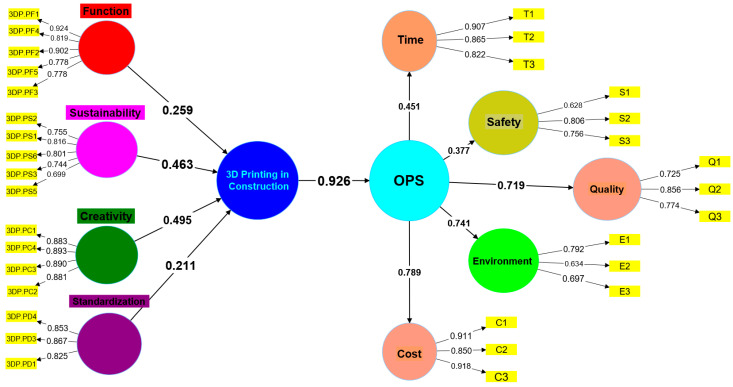
Structural model with path coefficients.

Evaluation of a measurement model includes estimating indicator consistency, combination reliability, extracted average variance (AVE), and discriminant validity. In accordance with PLS standards, this study employs a suitable weighting scheme, data measure, maximum iterations, abort criteria, and starting weights [94,102]. Figure 4 is an example of a structural equation model (SEM) addressing the hypothesis indicated in Figure 1. Based on the current literature, Table 1 and Table 2 define and classify the many 3D printing and OPS aspects of the idea. In accordance with Fraserhealth, Savalei and Wang and Rhemtulla [93,95,101], to increase the composite’s reliability and AVE, the threshold should be increased if eliminating indications with external loadings between 0.40 and 0.65 on the scale considerably improves the reliability and AVE. The variables assessing environmental stress with values below 0.5 were ruled out as insufficient to fulfil this criterion and, hence, were not further investigated. Table 4 shows that all constructs and their factors pass this test, and Figure 4 provides the external loadings for each variable in the simple measurement model. Loading factors greater than 0.6 were found for all of the external loads for items pertaining to the “Function”, “Sustainability”, “Standardization”, “Creativity”, and “Credibility” constructs, with the exception of 3DP.PS4 and 3DP.PR3, which had loadings of 0.569 and 0.573, respectively. However, the last construct, credibility, was excluded from the analysis because factor 3DP.PR1 was the only factor in the construct and that is not feasible in terms of construct modelling. Further strong relationships were found among all other constructs, indicating high composite reliability, Cronbach’s alpha and Ave. As can be seen in Table 4, all approved models had CRs greater than 0.70, indicating their reliability. When evaluating the convergent validity of model designs, AVE values over 0.50 are often tolerated. A concept has discriminant validity if it can be separated from other, comparable concepts by independent, external criteria. The construct’s selective validity demonstrates how its uniqueness enables it to catch occurrences that are poorly captured by other model conceptions.

The cross-loading criterion, the criterion of a ratio of correlations between heterotraits and monotraits (HTMT), and the criterion of independence are the three methods available for verifying discriminant validity. Ringle et al. [94] found that quantitative evidence for discriminative validity might be found by contrasting the square root of the AVE for each construct with correlations between any two constructs. The maximum allowable correlation between latent variables is the AVE’s square root. The results, shown in Table 5, demonstrate the discriminant validity of the measurement model. Assuming that the constructs were reliably assessed, the HTMT method offers a unique way to assess the discriminative validity of variance-based SEMs by studying the correlation between two constructs. In this study, the HTMT model was used to evaluate the discriminant’s efficacy. An HTMT value of 0.85–0.90 will do to show the difference between the two ideas. A score of less than 0.90 on the HTMT indicates conceptual similarity between the model structures, whereas a score of less than 0.85 indicates conceptual dissimilarity. All of the HTMT readings for the buildings under study are summarised in Table 6. Therefore, the ideas have enough discriminant validity. 

This study also used cross-loading criteria to prove discriminatory validity. According to Igolkina and Meshcheryakov and Zyphur and Pierides [96,98], the indicator loading on a particular latent construct is compared to the loading on all other latent constructs in a specific row to see whether there should be any discrepancy. So, their structures must have more indicator loading than standard structures. Table 7 shows that the indicator loads of all latent constructs exceed their cross-loadings on all other constructs (by row). The results show that most structures only have one dimension.

### 4.2. Measurement Model (Second Order Construct)

**Figure 5:**
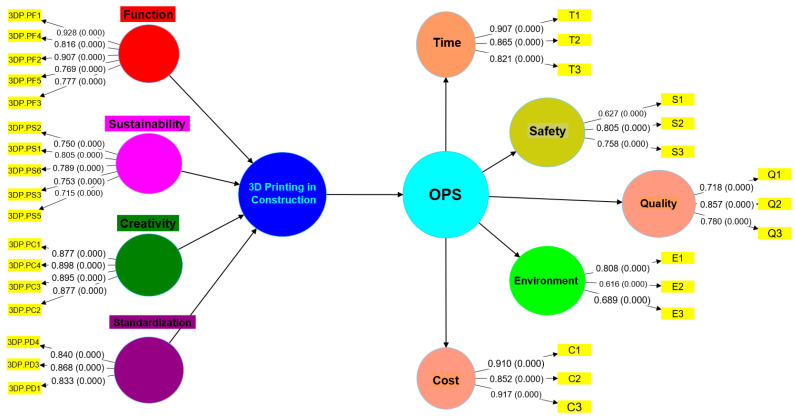
Bootstrapping analysis showing outer path indicating loading and *p*-values and inner weights with *p*-values.

Since the primary variables were all second-order latent factors, the bootstrap approach was used to assess the importance of each first-order latent variable. The 3D printing implementation project had a formative aspect, whereas the home-based OPS was more reflective. High levels of correlation between variables from various formative measuring methods are not usually immediately apparent. Jak and Cheung and Zyphur and Pierides [96,97] stated that there is a high potential for collinearity because of the close connection between the constituent elements. By quantifying the worth of 3D printing’s impact, we were able to examine the collinearity between the construct’s formative components. Internal VIF values were used to probe collinearity issues while handling reflective-formative second-order construct types. The components of the function stage, the sustainability stage, the creative stage, and the standardisation stage stood out, contributing to a relatively high standard route coefficient for 3D printing operations as indicated in Figure 5.

From Table 8, it was found that the creativity construct had the highest outer loading β of 0.495. For sustainability, function, and standardization, the outer loadings are 0.463, 0.259, and 0.211, respectively. The path significance values for all of the construction were less than 0.05 indicating high acceptability of results. The VIF values obtained were less than 3.5, indicating that each factor is contributing independently towards forming a higher-order construct. The formative second-order construct, therefore, contributed significantly to 3D printing in construction. Table 9 shows the bootstrapping statistics of reflective second-order constructs. Cost and environment have indicated the highest path coefficients of 0.789 and 0.741. For quality, safety, and time, the path coefficients were 0.719, 377, and 0.451, respectively. The evidence of significant and effective outcomes of reflective constructs indicates acceptable statistics regarding the impact of 3D printing in residential OPS.

### 4.3. Structural Model (Path Analysis)

Path analysis is an effective method for researchers interested in linear regression. Path analysis is the standard technique for scientific and social management. In addition, path analysis is the primary technique for investigating all intricate interrelationships concurrently. Much of the work in SEM analysis is performed with the use of a structural equation model [93,98]. The structural model may be used to analyse the interrelationships between the variables. Creating a structural equation model was the next crucial stage of the SEM analysis. According to Igolkina and Meshcheryakov and Shirgaokar [98,99], if the relationships between the variables are known, a structural model may be employed. The structural model provides a more thorough explanation of the connections between the various variables. The information demonstrates the connection between endogenous (inside) and exogenous (outside) variables. When assessing the aforementioned structural model, the overall model fit is of utmost importance, followed by the size, direction, and relevance of the estimated parameters. The research that used SEM to test the theory is shown in Figure 1. We utilised PLS-SEM to examine the impact of 3D printing adoption on OPS-related aspects based on the research framework provided by this model. Bootstrapping helped us evaluate the model’s null hypothesis and its significance [94,100]. By randomly resampling the original dataset, the bootstrapping method creates new samples of the same size as the original samples. How reliable the data is, how much room for error there is in the predicted route coefficients, and what degree of significance we can be sure of using. As the Table 10 shows, 3D printing has a major effect on OPS at residential construction projects. The study indicated that 3D printing had positive and statistically significant benefits on productivity in residential construction.

#### 4.3.1. Explanatory Power of the Structural Model

The results indicate not just strong covert validity and discriminant validity in the evaluative model but also high levels of reliability at the item level. According to Igolkina and Meshcheryakov and Savalei [93,98], to evaluate a model’s ability to explain data, one approach is to determine what percentage of the total variance in the dependent variable can be attributed to the model. Multiple R^2^ correlations between dependent variables inside a model may be determined using PLS. In PLS, the R^2^ value is said to be the same as in traditional regression. According to Shirgaokar and Zyphur and Pierides [96,99], the coefficient of determination (R^2^) is a statistical indicator of complete randomness. It was the independent factors inside the dependent variable that provided the reason for this. A higher R^2^ value indicates a more robust structural model. Table 11 displays the results of studies on regression. With an updated R^2^ of 0.9, we see that the exogenous latent variable (3D printing in construction) can explain 90% of the variation in project performance, making it the most important dependent variable in our model. Larsson et al. and Tarka [92,100] stated that whether or not an independent construct has a significant impact on the dependent constructs may be inferred by analysing the change in R^2^ after eliminating the construct from the model. The effect size (f^2^) measurement is therefore carried out in accordance with Equation (1) [101]. A high effect size of 1.33 was observed.

The computation of the effect size is,

(1) $$Effect\;Size=f^{2}=\frac{R^{2}include-R^{2}exclude}{1-2R^{2}exclude}$$

$f^{2}=0.02\;\to Moderate;f^{2}=0.15\;\to Medium;f^{2}=0.35\;\to High$

#### 4.3.2. Predictive Relevance of the Structural Model

Predictive validity assessment is an essential part of any structural model. The blind method was used to verify the redundancy estimates obtained by cross-validation for each dependent variable. Statistical analysis of the data revealed that the Q^2^ scores had a predictive value (0.919 for project performance) above zero, suggesting that the independent construct had predictive relevance for the dependent construct under consideration. Table 12 shows that Q^2^ is greater than 0. It is reasonable to assume that the model has a very good predictive ability.

#### 4.3.3. Analysis of Performance Matrix Importance

PLS-SEM provides empirical evidence for the relative significance of an independent variable in explaining the dependent variable. According to Igolkina and Meshcheryakov [98], in deciding amongst these potential managerial measures, it is crucial to consider both their relevance and their performance. By analysing the model’s overall effects (importance), as well as the mean value for latent variable scales, key areas for improving management operations (or the model’s focus) may be identified (performance). The success of the 3D printing in this investigation relied on importance-performance map analysis (IPMA). Table 13 presents the overall performance and importance of latent exogenous variables in 3D printing in construction.

## 5. Discussion

Success rates on Malaysian residential projects can be greatly improved by incorporating 3D printing. There is a good basis for investigating connections between the models thanks to the statistical data gathered by testing the models using the modified SEM approach.

The function formative construct includes 3DP.PF1: “Making customized residential structures available to the wider market”, 3DP.PF2: “Simpler and more efficient installation”, 3DP.PF3: “Increased rate of construction efficiency”, 3DP.PF4: “Accuracy that is far higher than before”, and 3DP.PF5: “Quick and easy prototypes”. Three-dimensional printing has been discovered to be a simpler and more efficient installation method in Malaysia’s residential sector. It is advantage number two in providing effective outcomes for the residential sector of Malaysia, as ultimately it can help to save a lot of time and reduce costs. El-Sayegh et al. and Yang et al. [7,41] also indicate a similar behaviour where 3D printing is found to be significant for housing projects in terms of effective installation. According to Kazemian and Khoshnevis and Poluektova [43,55], the other factors have a moderately similar impact on Malaysia’s housing industry. The most important factor from a functional standpoint is the customization of residential structures, which is consistent with current research, which classifies 3D construction printing as the most highly customizable technology currently available in the world for the construction industry. The different behaviour is observed in the sense of an overall combination of factors, because ultimately it is indicated that the function construct contributes significantly to the 3D printing impact, with customisation and efficient installation being the most notable factors.

The sustainability formative construct includes, 3DP.PS1: “Enables the design and construction of environmentally responsible buildings”, 3DP.PS2: “The recycling of trash into a new product”, 3DP.PS3: “The printing technique will reduce waste, minimizing production/environmental construction’s effect”, 3DP.PS5: “Enable and provide advanced healthcare”, and 3DP.PS6: “Reduced human impact”. The sustainability construct clearly shows that 3D construction printing will have a positive impact on the environment of the residential construction sector while also being critical in terms of making the residential sector sustainable. The observed indication of environmental sustainability is in accordance with Bergeron et al. and Mascarenhas and Makhoul [54,56], where it is regarded as the main advantage of adopting 3D construction printing in the housing industry. It is also a notable factor from the outcomes that the human impact is being reduced with the help of 3D construction printing, which is effectively identified by M. H. Ali et al. and Z. Xu et al. [69,80], where 3D construction printing is found to be more error-free and reliable in terms of promoting sustainability in the housing industry. The primary distinction in this regard is the overall sustainability perspective, in which recycling is also identified as important for Malaysia’s residential sector mobile and is also indicating a positive impact on worker health and safety outcomes.

The standardisation formative construct includes, 3DP.PD1: “Fewer logistical procedures and waste”, 3DP.PD2: “A shorter supply chain and a faster design cycle”, 3DP.PD3: “Reduce human mistakes”, and 3DP.PD4: “Evaluate ideas generated during brainstorming considering the intended outcomes”. It is indicated by the standardization construct that there is a significant improvement in the reduction of human mistakes while adopting 3D construction printing in the residential construction sector of Malaysia. The indication is strong in the sense of M. H. Ali et al. and Han et al. [70,74], where it is highly regarded as a prominent technology that can minimise errors in construction as a significant level of automation is involved. The observed behaviour is also indicating that it is also important in terms of generating new ideas in construction, which is truly evident from Adaloudis and Bonnin Roca [79] in other parts of the world where 3D printing is found to be highly innovative for model housing architecture. The outcomes provide a unique way of understanding the impact of 3D printing in the residential construction sector of Malaysia, which is different in the sense of an effective combination of factors promoting a shorter supply chain, error reduction, and improved idea generation.

The creativity formative construct includes, 3DP.PC1: “Provide Innovative solutions”, 3DP.PC2: “Modelling architectural construction”, 3DP.PC3: “Permits more geometric flexibility when designing buildings that would not be achievable otherwise”, and 3DP.PC4: “Flexible design and brand improvement”. The flexible design and brand improvement discount are highly significant for the residential construction sector of Malaysia, which is critical in the sense of Aghimien et al. and Skoratko and Katzer [12,72] where 3D construction printing is already regarded as a prominent technology for the housing sector. The ranking of factors indicated in the creativity construct is entirely different in the sense of Abdalla et al., Adaloudis and Bonnin Roca, and Sun et al. [71,73,79], which is unique in the context of Malaysia’s residential sector and their efforts to promote the adoption of 3D construction printing. It is also indicated by creatively constructing that, with the help of 3D construction printing, the possible applications of improving geometric flexibility and implementing innovative solutions become very high. The observed behaviour is in accordance with the grassing research while also indicating differentiation, which is fully linked with the different states of the Malaysian residential construction sector.

The time reflective construct includes, T1: “On time project delivery”, T2: “Timely project delivery involving variations”, and T3: “Timely availability of resources needed for project completion”. It is evident that 3D printing is directly improving the on-time project delivery aspect of ops, which shatters the argument provided by existing research in which time is regarded as one of the critical factors. The indication is also so strong in terms of promoting the timely availability of resources required for project delivery, and the study is providing effective insights in accordance with Bedarf et al., Plarre et al., and Lam et al. [62,63,64] on 3D printing in the housing industry. The impact condition is completely different from Besklubova et al. and Ting et al. [38,60], in which time was regarded as important, but project delivery is most critical for Malaysian residential construction projects if they adopt 3D construction printing in the future. It is a strong indication that 3D printing for construction will improve the project timeline.

The cost reflective construct includes, C1: “Profit margin improvement”, C2: “Cash flow enhancement”, and C3: “Reduction in variable costs”. It is found that profit margin improvement is the ultimate benefit of adopting 3D construction printing, and with respect to Khoshnevis, Pan et al., and Shahzad et al. [55,57,59], it is also found to be reliant on making the construction project successful. The cost of production is already prone to being one of the critical advantages of 3D construction printing, as is evident from the outcomes of this study. It is in accordance with the concept that there is any improvement in sustainability with the help of cost improvement, while it is different only in the sense of global research, where the Malaysian residential construction sector is giving more prominence to profit margin improvement. The observed behaviour is indicating significant implications for the residential construction sector in Malaysia, where it will be necessary to adopt 3D construction printing in terms of cost reduction and promoting OPS.

The quality reflective construct includes, Q1: “All specifications are met”, Q2: “All resources are available for required quality delivery”, and Q3: “Delivering project with compliance to equipment and raw material quality”. The availability of resources is critically linked with the quality delivery of overall projects involving 3D construction printing, and this is in accordance with existing research in terms of identifying the quality parameters as critical outcomes of implementing 3D construction printing in the housing industry. It is also an effective indication of different research outcomes because, in the Malaysian residential construction sector, there is more importance given by participants to resource management, which is totally linked with implementing 3D construction printing in accordance with Bedarf et al. and Hoffmann et al. [63,76]. This behaviour is attributable to the concept of overall compliance with raw material quality as well as maintaining the quality delivery of projects, which is in accordance with existing research indicating successful outcomes of projects.

The environmental protection reflective construct includes, E1: “Sustainable logistics with reduced wastage of materials”, E2: “Environmental protection objectives and standards satisfied”, and E3: “Reduced energy consumption with a reduction in net embodied carbon”. The reduction in waste generation of materials with sustainable logistics is identified to be the highest significant sub-factor that is critically improving the environment and ultimately providing benefits to overall project success. It is the reason that the positive implications of 3D construction printing are linked with maximizing the environmental benefits. Further, when the environmental standards are met in the residential project, it ultimately improves the rate of success and also allows the 3D printing technology to be adopted as a new technology for further development with environmental protection. Similar is the case with the reduction in energy consumption, as the project completed by 3D construction printing will have benefits for the environment and ultimately reduce embodied carbon. As indicated by Cascone et al. and Gomez et al. [45,49], significant positive implications exist in terms of improving the environment with the help of 3D construction printing. The different results observed from the analysis where a reduction in waste of materials can be regarded as a critical factor that will ultimately improve environment protection. It is especially relevant in the residential construction environment of Malaysia, where a significant challenge has always been to reduce the waste of construction activities, which is ultimately susceptible to the effective relationship between 3D construction printing and the environmental production aspect of overall project success [34,45,68].

The public health and safety reflective construct includes, S1: “Sustainable physical well-being of workers”, S2: “Increased security and safety, less dependency on human resources”, and S3: “Effective public health hazards management on worksite with technology”. It is the unique quality of 3D construction printing observed from analysis that helps reduce human resource dependency, which ultimately improves safety on the work site. Na et al., Kazemain et al., and Faham et al. [5,9,89] have shown significant evidence of increasing public health safety when automation is involved, and a similar effect is observed in the case of adopting theory-based construction printing. That is indicated in terms of improving the sustainable physical well-being of workers as well as providing effective public health hazard management on the job side with automatic technology. However, different behaviour is observed, indicating the greater importance of increasing worker security and safety with the help of 3D construction printing. Positive implications are observed as 3D construction printing contributes significantly to public health and safety aspects of overall project success and can be highly appropriate for residential construction environments in Malaysia.

All five dimensions of the overall project success have contributed significantly to indicating the possible benefits of using 3D construction printing in the residential construction sector of Malaysia. The cost–time benefits are clearly indicated, which ultimately maximise the success rate of projects while ensuring further implications for increasing safety for workers and maintaining efficient public health standards. Furthermore, an incorrect implication was observed with regard to the environment, as a result of which construction 3D printing ultimately helps to reduce waste, which supports overall project success. It is the reason that 3D construction printing is positively linked with increasing public health and environmental compliance. For the practical improvement of public health and the production of the environment on residential construction project work sites, it is important that 3D construction printing is adopted by the use of the latest technology. Even at a smaller scale, 3D construction printing can revamp the overall residential construction and sea of Malaysia, which can ultimately contribute to reducing the frequency of injuries and allowing the workers to ultimately protect the environment, which is one of the critical hazards contributing to global climate change. The creativity aspects are more prominent in the analysis, which indicates possible ways of innovation that can further improve the efficiency of technology in maintaining overall project success in residential construction projects in Malaysia.

## 6. Conclusions

To verify the links between 3D printing and OPS (cost, time, quality, public health safety, and environmental protection) in Malaysian home construction, a PLS-SEM study was conducted. Specialists in the building trade provided the evidence necessary to verify the direct and indirect channels as essential to the structural model. Furthermore, both direct and indirect correlations between activity components and covariates were confirmed. Based on the findings, 3D printing may be used to save costs and boost the success rate of any project. 

Three-dimensional printing improves Malaysian house construction by reducing time and cost. Furthermore, 3D printing is vital for housing developments. Additional factors may impact Malaysia’s housing market in the future, but 3D construction printing will always help to improve all scope dimensions of a project. Residential building modification is functionally important. Three-dimensional printing is the most adaptable building method, according to a study. Customisation and successful installation cause varying behaviour.

The sustainability build shows that 3D construction printing will favourably impact the home building sector’s environment. According to a study, 3D printing in houses promotes environmental sustainability. Three-dimensional building printing reduces human impact and promotes sustainability in the housing industry, according to research. Sustainability-wise, recycling is important for Malaysia’s mobile home sector and worker health and safety.

Standardisation in Malaysia’s house-building sector eliminates human mistakes. According to research, it may decrease construction errors via automation. Three-dimensional printing is revolutionising model home design in different parts of the world, as shown by the observed behaviour. The findings provide a new way to measure 3D printing’s impact on Malaysia’s homebuilding industry, which encourages a shorter supply chain, mistake reduction, and idea creation.

Flexible design and improvement are significant for Malaysia’s residential building industry, as 3D printing is a prominent housing technology. Unique in Malaysia’s home industry, the creative construct ranks components by worldwide demand to stimulate 3D building printing. Three-dimensional printing may boost geometric flexibility and generate unique solutions. 

It may be enhanced by cutting expenses; however, research suggests that the Malaysian residential construction market emphasises profit margins. Three-dimensional printing will be required to cut costs and enhance OPS in Malaysia’s homebuilding industry. The availability of resources affects the quality of 3D construction printing projects, which is consistent with past research stressing quality criteria as critical consequences of adopting 3D printing in the housing industry. In Malaysia’s residential building business, resource management is linked to 3D printing. The behaviour is related to compliance with raw material quality and quality project delivery, which is consistent with excellent project outcomes.

It has been noted by academics that the methods and tactics for implementing 3D printing have been understudied. While many have looked at the effects of 3D printing on OPS, most have neglected to address the true causes of this condition. The present research fills a gap in the existing literature by examining the relationship between 3D printing and OPS. The primary gain from this research is a stronger foundation for engineering project management in the residential construction industry in Malaysia, specifically in one chosen sector. Second, this research paves the way for more research by demonstrating the tangible benefits to all dimensions of OPS brought about by the widespread use of 3D printing. This new information may encourage further study of 3D-printed residential developments.

And lastly, despite Malaysia’s standing as a developed nation with strong development in construction, most 3D printing legislation and plans have not moved beyond the planning phases. Three-dimensional printing has gained popularity in Malaysia as a rapid and cost-effective manufacturing technology. However, its widespread use raises concerns about its potential impact on public safety and the environment. One of the major limitations of 3D printing is the lack of regulations and standards governing the production and use of 3D-printed products, which can pose risks to public safety. The use of low-quality or untested materials in 3D printing can result in weak, defective, or hazardous products that can harm users. In addition, the disposal of 3D-printed waste, which can be non-biodegradable and contain toxic materials, poses a threat to the environment. Another limitation is the energy consumption associated with 3D printing, which can contribute to carbon emissions and climate change. Overall, the adoption of 3D printing in Malaysia should be accompanied by proper regulations and guidelines to ensure public safety and minimise its impact on the environment. No studies have looked at the outcomes of employing 3D printing in the OPS, and there is a gap in research on the issue in the Malaysian residential construction industry. This kind of study is crucial to the development and general use of 3D printing in the field. This study provides the groundwork for 3D printing to become widely used in the Malaysian construction industry. Project practitioners, such as building owners and contractors, may benefit from the article’s guidance since it explains how they can utilise 3D printing to improve the public health safety, environment, time, cost, and quality of their projects. This study has the potential to impact the success of future residential projects by assisting project stakeholders in accepting and executing 3D printing with a focus on OPS at every stage of the project’s existence.

## Figures and Tables

**Table 1:** Three-dimensional printing impact factors evident from existing literature.

3DP Phases	Assigned Code	Activities	References
Function	3DP.PF1	Making customised residential structures available to the wider market	[63,64,65]
	3DP.PF2	Simpler and more efficient installation	[60,62]
	3DP.PF3	Increased rate of construction efficiency	[49,66]
	3DP.PF4	Accuracy that is far higher than before	[52,67]
	3DP.PF5	Quick and easy prototypes	[53,68]
Sustainability	3DP.PS1	Enables the design and construction of environmentally responsible buildings	[69,70]
	3DP.PS2	The recycling of trash into a new product	[12,71]
	3DP.PS3	The printing technique will reduce waste, minimising production/environmental construction’s effect	[71,72]
	3DP.PS4	Reduce the usage of formwork	[70,73,74]
	3DP.PS5	Enable and provide advanced healthcare	[75,76]
	3DP.PS6	Reduced human impact	[38,62]
Standardisation	3DP.PD1	Fewer logistical procedures and waste	[72,76,77]
	3DP.PD2	A shorter supply chain and a faster design cycle	[73,74,78]
	3DP.PD3	Reduce human mistakes	[71,72]
	3DP.PD4	Evaluate ideas generated during brainstorming considering the intended outcomes	[73,79]
Creativity	3DP.PC1	Provide innovative solutions	[80,81]
	3DP.PC2	Modelling architectural construction	[69,81]
	3DP.PC3	Permits more geometric flexibility when designing buildings that would not be achievable otherwise	[12]
	3DP.PC4	Flexible design and brand improvement	[70,74]
Credibility	3DP.PR1	Increased value in global market	[79]
	3DP.PR2	Bonding for accuracy and complexity	[71,72,73]
	3DP.PR3	Supports unusual and green designs	[60,61]
	3DP.PR4	Integration with other components	[75,76]

**Table 2:** Factors leading to overall project success.

OPS Factors	Code	Sub-Factors	References
Time management	T1	On time project delivery	[61,63]
	T2	Timely project delivery involving variations	[24,27,28]
	T3	Timely availability of resources needed for project completion	[72,81]
Cost management	C1	Profit margin improvement	[74,79]
	C2	Cash flow enhancement	[58,60]
	C3	Reduction in variable costs	[55,56,59]
Quality management	Q1	All specifications are met	[58,60,61]
	Q2	All resources are available for required quality delivery	[38,60]
	Q3	Delivering project with compliance to equipment and raw material quality	[55,56]
Public health and safety	S1	Sustainable physical well-being of workers	[82,83]
	S2	Increased security and safety, less dependency on human resources	[82,83]
	S3	Effective public health hazards management on worksite with technology	[84,85,86]
Environmental protection	E1	Sustainable logistics with reduced wastage of materials	[87,88,89]
	E2	Environmental protection objectives and standards satisfied	[90,91]
	E3	Reduced energy consumption with reduction in net embodied carbon	[87,88,91]

**Table 3:** Rotated component matrix with reliability statistics.

Variables	Component					Cronbach’s Alpha
	1	2	3	4	5	
3DP.PF1	0.872					0.894
3DP.PF2	0.842					
3DP.PF3	0.770					
3DP.PF4	0.749					
3DP.PF5	0.736					
3DP.PS1		0.784				0.836
3DP.PS2		0.750				
3DP.PS3		0.707				
3DP.PS4		0.699				
3DP.PS5		0.654				
3DP.PS6		0.649				
3DP.PC1			0.864			0.910
3DP.PC2			0.861			
3DP.PC3			0.802			
3DP.PC4			0.781			
3DP.PD4				0.842		0.806
3DP.PD1				0.753		
3DP.PD3				0.720		
3DP.PR3					0.649	0.701
3DP.PR1					0.617	
Eigen value	3.917	3.411	3.389	2.592	2.139	
%Variance	15.066	13.121	13.034	9.969	8.228	

Extraction Method: principal component analysis. Rotation method: Varimax with Kaiser normalization. Variables 3DP.PR2, 3DP.PR4 and 3DP.PD2 deleted from EFA because of cross loading and loading less than 0.6.

**Table 4:** Construct initial loadings, modified loadings, and reliability statistics.

Construct	Assigned Code	Initial Loadings	Modified Loadings	Cronbach’s Alpha	Composite Reliability	AVE
Function	3DP.PF1	0.924	0.924	0.896	0.924	0.71
	3DP.PF2	0.902	0.902	-	-	-
	3DP.PF3	0.778	0.778	-	-	-
	3DP.PF4	0.819	0.819	-	-	-
	3DP.PF5	0.780	0.780	-	-	-
Sustainability	3DP.PS1	0.816	0.811	0.821	0.875	0.584
	3DP.PS2	0.755	0.766	-	-	-
	3DP.PS3	0.744	0.737	-	-	-
	3DP.PS4	0.564	Deleted	-	-	-
	3DP.PS5	0.699	0.704	-	-	-
	3DP.PS6	0.801	0.796	-	-	-
Standardisation	3DP.PD1	0.825	0.825	0.806	0.885	0.719
	3DP.PD3	0.867	0.867	-	-	-
	3DP.PD4	0.853	0.853	-	-	-
Creativity	3DP.PC1	0.883	0.883	-	-	-
	3DP.PC2	0.881	0.881	-	-	-
	3DP.PC3	0.890	0.890	-	-	-
	3DP.PC4	0.893	0.893	-	-	-
Credibility	3DP.PR1	0.867	Excluded	-	-	-
	3DP.PR3	0.573	Deleted	-	-	-
Cost	C1	0.911	0.911	0.873	0.922	0.798
	C2	0.850	0.850	-	-	-
	C3	0.918	0.918	-	-	-
Time	T1	0.907	0.907	0.834	0.900	0.755
	T2	0.865	0.865	-	-	-
	T3	0.822	0.822	-	-	-
Quality	Q1	0.725	0.725	0.701	0.829	0.626
	Q2	0.856	0.856	-	-	-
	Q3	0.774	0.774	-	-	-
Safety	S1	0.628	0.628	0.699	0.776	0.638
	S2	0.806	0.806	-	-	-
	S3	0.756	0.756	-	-	-
Environment	E1	0.792	0.792	0.692	0.752	0.575
	E2	0.634	0.634	-	-	-
	E3	0.697	0.697	-	-	-

**Table 5:** Correlations of latent variables and discriminant validity.

	Cost	Creativity	Environment	Function	Quality	Safety	Standardization	Sustainability	Time
Cost	0.893								
Creativity	0.788	0.887							
Environment	0.728	0.703	0.711						
Function	0.353	0.331	0.701	0.843					
Quality	0.205	0.197	0.483	0.399	0.787				
Safety	0.28	0.282	0.354	0.397	0.356	0.734			
Standardisation	0.252	0.253	0.326	0.378	0.257	0.563	0.848		
Sustainability	0.19	0.184	0.561	0.444	0.752	0.33	0.258	0.764	
Time	0.336	0.316	0.707	0.777	0.378	0.381	0.383	0.429	0.865

**Table 6:** HTMT statistics.

	Cost	Creativity	Environment	Function	Quality	Safety	Standardization	Sustainability	Time
Cost									
Creativity	0.107								
Environment	0.699	0.641							
Function	0.39	0.355	0.092						
Quality	0.281	0.26	0.66	0.505					
Safety	0.384	0.379	0.643	0.518	0.547				
Standardisation	0.298	0.292	0.498	0.439	0.33	0.716			
Sustainability	0.233	0.218	0.731	0.518	0.266	0.462	0.308		
Time	0.38	0.347	0.144	0.125	0.489	0.51	0.46	0.514	

**Table 7:** Statistically determined cross-loadings for discriminant validity.

	Cost	Creativity	Environment	Function	Quality	Safety	Standardisation	Sustainability	Time
C1	**0.911**	0.238	0.792	0.375	0.197	0.233	0.245	0.195	0.359
C2	**0.85**	0.216	0.51	0.197	0.129	0.276	0.238	0.118	0.184
C3	**0.918**	0.245	0.645	0.363	0.217	0.244	0.197	0.192	0.347
3DP.PC1	0.238	**0.883**	0.792	0.375	0.197	0.233	0.245	0.195	0.359
3DP.PC2	0.216	**0.881**	0.645	0.363	0.217	0.244	0.197	0.192	0.347
3DP.PC3	0.245	**0.89**	0.51	0.197	0.129	0.276	0.238	0.118	0.184
3DP.PC4	0.815	**0.893**	0.519	0.215	0.144	0.251	0.216	0.139	0.209
E1	0.711	0.671	**0.883**	0.375	0.197	0.233	0.245	0.195	0.359
E2	0.144	0.139	**0.634**	0.438	0.599	0.172	0.115	0.555	0.436
E3	0.276	0.259	**0.697**	0.619	0.331	0.392	0.356	0.377	0.665
3DP.PF1	0.351	0.331	0.621	**0.924**	0.372	0.347	0.339	0.406	0.907
3DP.PF2	0.361	0.339	0.6	**0.902**	0.373	0.388	0.33	0.398	0.847
3DP.PF3	0.253	0.234	0.525	**0.778**	0.332	0.295	0.27	0.364	0.667
3DP.PF4	0.276	0.259	0.697	**0.819**	0.331	0.392	0.356	0.377	0.765
3DP.PF5	0.226	0.211	0.506	**0.778**	0.264	0.233	0.297	0.32	0.722
Q1	0.045	0.036	0.357	0.318	**0.725**	0.19	0.195	0.262	0.305
Q2	0.194	0.183	0.398	0.334	**0.856**	0.29	0.254	0.182	0.319
Q3	0.244	0.245	0.386	0.289	**0.774**	0.363	0.145	0.257	0.267
S1	0.24	0.237	0.295	0.391	0.286	**0.628**	0.625	0.222	0.383
S2	0.181	0.187	0.247	0.238	0.278	**0.806**	0.152	0.262	0.219
S3	0.175	0.178	0.211	0.195	0.188	**0.756**	0.118	0.182	0.188
3DP.PD1	0.24	0.237	0.295	0.391	0.286	0.628	**0.825**	0.257	0.383
3DP.PD3	0.23	0.219	0.308	0.296	0.202	0.408	**0.867**	0.222	0.303
3DP.PD4	0.16	0.179	0.217	0.262	0.151	0.367	**0.853**	0.166	0.277
3DP.PS1	0.045	0.036	0.357	0.318	0.262	0.19	0.195	**0.816**	0.305
3DP.PS2	0.144	0.139	0.634	0.438	0.182	0.172	0.115	**0.755**	0.436
3DP.PS3	0.194	0.183	0.398	0.334	0.257	0.29	0.254	**0.744**	0.319
3DP.PS5	0.244	0.245	0.386	0.289	0.222	0.363	0.145	**0.699**	0.267
3DP.PS6	0.095	0.095	0.373	0.316	0.262	0.238	0.262	**0.801**	0.311
T1	0.351	0.331	0.621	0.924	0.182	0.347	0.339	0.406	**0.907**
T2	0.276	0.259	0.697	0.819	0.331	0.392	0.356	0.377	**0.865**
T3	0.226	0.211	0.506	0.778	0.264	0.233	0.297	0.32	**0.822**

**Table 8:** Bootstrapping statistics for formative constructs.

Path	β	SE	t-Values	*p*-Values	VIF
Creativity → 3D printing	0.495	0.034	15.344	<0.001	1.148
Function → 3D printing	0.259	0.032	9.431	<0.001	1.453
Standardization → 3D printing	0.211	0.024	9.298	<0.001	1.202
Sustainability → 3D printing	0.463	0.033	12.933	<0.001	1.262

**Table 9:** Bootstrapping statistics for reflective constructs.

Path	β	SE	t-Values	*p*-Values
OPS → cost	0.789	0.036	22.640	<0.001
OPS → quality	0.719	0.059	11.658	<0.001
OPS → time	0.451	0.054	8.258	<0.001
OPS → safety	0.377	0.058	6.483	<0.001
OPS → environment	0.741	0.031	23.804	<0.001

**Table 10:** Path validation between 3D printing and OPS.

Path	β	SE	t-Values	*p*-Values
3D printing → OPS	0.92	0.006	48.3	<0.001

**Table 11:** R-square determination.

Endogenous Latent Variable	R^2^	Adjusted R^2^	Explained Size
Project success	0.9	0.9	Highly predictive

**Table 12:** Predictive relevance.

Endogenous Latent Variable	Predict-Q^2^
Project success	0.919

**Table 13:** Overall effects and importance of 3D printing.

Predictor	Importance	Performance
3D printing	1.934	53.213

## Data Availability

The data are not publicly available due to privacy restrictions.

## Abbreviations

3DP	3D printing
OPS	Overall project success
SEM	Structure equation modelling
IPMA	Importance-performance map analysis
ANN	Artificial neural network
EFA	Exploratory factor analysis
